# Patterns and drivers of plant carbon, nitrogen, and phosphorus stoichiometry in a novel riparian ecosystem

**DOI:** 10.3389/fpls.2024.1354222

**Published:** 2024-04-08

**Authors:** Lei Wang, Muhammad Arif, Jie Zheng, Changxiao Li

**Affiliations:** ^1^ Key Laboratory of Eco-Environments in the Three Gorges Reservoir Region (Ministry of Education), School of Life Sciences, Southwest University, Chongqing, China; ^2^ Chongqing Key Laboratory of Plant Ecology and Resources Research in the Three Gorges Reservoir Region, School of Life Sciences, Southwest University, Chongqing, China; ^3^ Biological Science Research Center, Academy for Advanced Interdisciplinary Studies, Southwest University, Chongqing, China

**Keywords:** ecological stoichiometry, life forms, riparian plants, hydrological change, flooding intensity, three gorges reservoir

## Abstract

Carbon (C), nitrogen (N), and phosphorus (P) stoichiometry serve as valuable indices for plant nutrient utilization and biogeochemical cycling within ecosystems. However, the allocation of these nutrients among different plant organs and the underlying drivers in dynamic riparian ecosystems remain inadequately understood. In this study, we gathered plant samples from diverse life forms (annuals and perennials) and organs (leaves, stems, and roots) in the riparian zone of the Three Gorges Reservoir Region (TGRR) in China—a novel ecosystem subject to winter flooding. We used random forest analysis and structural equation modeling to find out how flooding, life forms, plant communities, and soil variables affect organs C, N, and P levels. Results showed that the mean concentrations of plant C, N, and P in the riparian zone of the TGRR were 386.65, 19.31, and 5.27 mg/g for leaves respectively, 404.02, 11.23, and 4.81 mg/g for stems respectively, and 388.22, 9.32, and 3.27 mg/g for roots respectively. The C:N, C:P and N:P ratios were 16.15, 191.7 and 5.56 for leaves respectively; 26.98, 273.72 and 4.6 for stems respectively; and 16.63, 223.06 and 4.77 for roots respectively. Riparian plants exhibited nitrogen limitation, with weak carbon sequestration, low nutrient utilization efficiency, and a high capacity for nutrient uptake. Plant C:N:P stoichiometry was significantly different across life forms and organs, with higher N and P concentrations in leaves than stems and roots, and higher in annuals than perennials. While flooding stress triggered distinct responses in the C, N, and P concentrations among annual and perennial plants, they maintained similar stoichiometric ratios along flooding gradients. Furthermore, our investigation identified soil properties and life forms as more influential factors than plant communities in shaping variations in C:N:P stoichiometry in organs. Flooding indirectly impacts plant C:N:P stoichiometry primarily through alterations in plant community composition and soil factors. This study underscores the potential for hydrologic changes to influence plant community composition and soil nutrient dynamics, and further alter plant ecological strategies and biogeochemical cycling in riparian ecosystems.

## 1 Introduction

Carbon (C), nitrogen (N), and phosphorus (P) are indispensable elements in plant growth (Chang et al., 2022). C serves as the fundamental energy supplier in ecosystems (Wang et al., 2021), while N and P play crucial roles in electron transfer during respiration and act as key limiting factors for primary production (Tang et al., 2018; Zhang et al., 2018). Variations in plant C:N ratios can impact microbial mineralization rates, thereby influencing the global carbon cycle (Elser et al., 2008; Spohn et al., 2023). Additionally, nutrient limitations may shift from N to P as plant N:P ratios increase, ultimately leading to changes in vegetation composition and ecosystem function (Gusewell, 2004; Sardans et al., 2012). In riparian ecosystems, frequent flooding results in plant organ mortality, exacerbating soil nutrient loss, and altering soil nutrient ratios (Li et al., 2019; Zheng et al., 2021b). Under conditions of flood stress and nutrient co-limitation, the physiological processes of C, N, and P in riparian plants are constrained, including photosynthesis and nutrient mineralization (Ye et al., 2020; Cao et al., 2022). Inundation-induced changes in plant composition and soil properties may impact nutrient interactions in riparian plant-soil systems (Yu et al., 2019; Ye et al., 2020). Therefore, investigating plant C:N:P stoichiometry patterns and their drivers can enhance the insights into plant adaptation strategies and ecosystem functioning in dynamic riparian habitats.

The response of plant ecological stoichiometry to environmental gradients has been a focal point in recent years (Han et al., 2005; Sardans et al., 2021). Studies conducted at local, regional, global, and gene-to-ecosystem scales, based on field experiments or meta-analysis, have contributed to our understanding (Elser et al., 2008; Han et al., 2011; Yu et al., 2021; Cao et al., 2022). However, most prior studies have concentrated on agricultural, desert, grassland, and forest ecosystems (Xiong et al., 2021; Zhang et al., 2021a). By contrast, for dynamic riparian ecosystems, a comprehensive analysis of plant C:N:P stoichiometry remains lacking until now, partly due to the lack of comprehensive data sets. For instance, several studies analyzed the patterns of plant C:N:P stoichiometry in riparian ecosystems, but either considered only a few species or focused only on plant leaves, ignoring plant stems and roots and their influences (Huang et al., 2019; Zhou et al., 2021; Ding et al., 2022; Jing et al., 2022). This bias might be attributed to sampling difficulties or higher labor costs (Xing et al., 2022), limiting our comprehensive understanding of plant adaptation strategies. Several studies have indicated that organs such as roots can more accurately determine plant nutrient limitation traits than leaves (Tian et al., 2018; Yin et al., 2021). Nutrient partitioning among different plant organs reflects their trade-offs in accessing aboveground and belowground resources (Hu et al., 2021). Riparian plants must effectively allocate limited resources among organs to withstand the combined stresses of inundation and nutrient limitation (Jing et al., 2022). Therefore, gaining further insights into the elemental stoichiometric characteristics of different organs under varying inundation conditions is crucial for understanding riparian plant adaptation strategies.

Plant community characteristics and abiotic factors, such as geographical, climatic, and soil factors, exert varying degrees of influence on plant elemental stoichiometry in terrestrial ecosystems (Han et al., 2011; Chang et al., 2022). Plant elemental stoichiometry patterns exhibit distinctions across different organs and life forms due to functional variations in the growth process of these organs (He et al., 2015b; Yin et al., 2021; Xing et al., 2022). The limiting element stability hypothesis posits (Han et al., 2011) that more active organs, with a higher demand for essential elements (e.g., N and P), may exhibit greater N:P stoichiometric homeostasis (Zhang et al., 2018). Evidence suggests that flooding can lead to wetland plants experiencing co-limited by multiple elements, such as N and P (Zhang et al., 2021b; Cao et al., 2022). Moreover, life forms significantly influence plant C/N/P stoichiometry (Reich and Oleksyn, 2004; Xing et al., 2022), even within herbaceous plants (Lu et al., 2023). For instance, perennial plants, despite being herbaceous, can store organic carbon and possess higher nutrients by allocating more resources underground compared to annual plants (Poppenwimer et al., 2023). Additionally, numerous studies have affirmed that plant C/N/P ratios are affected by soil properties (Zhang et al., 2017; Chang et al., 2022). Leaf C/N/P ratios, for example, correlate with soil bulk density and pH (Zhang et al., 2019), while below-ground organs are more susceptible to soil nutrient variations (Yin et al., 2021). Soil properties further regulate plant elemental stoichiometry by impacting plant community characteristics, including community cover, height, and diversity (Wang et al., 2020; Ning et al., 2021; Yu et al., 2021; Zhang et al., 2021a). Previous studies have suggested that multiple factors may collaboratively shape plant elemental concentrations and stoichiometric patterns.

The Three Gorges Reservoir Riparian (TGRR), formed by the full operation of the Three Gorges Dam in 2010 (Zheng et al., 2021a), constitutes a significant hydraulic project on the Yangtze River in China. The TGRR represents a recently established ecosystem, experiencing a 30 m depth of inundation annually during the winter compared to during the summer or growing season (Zheng et al., 2021b). This setting serves as a distinctive natural laboratory for investigating inundation impacts on plant elemental stoichiometry. Riparian vegetation in TGRR plays a crucial role in sustaining river health, ecological balance, and overall sustainable development within the region and the Yangtze River Basin (Arif et al., 2023). Given the sensitivity of riparian vegetation to hydrological changes, its response to alterations in water flow has garnered increasing global attention (Lozanovska et al., 2020; Su et al., 2020). Considering the substantial rise in river damming worldwide and the anticipated increase in unpredictable precipitation events (Grill et al., 2019), the nutrient stoichiometry patterns of riparian plants could undergo significant transformations due to exposure to varying degrees of inundation, potentially differing from those observed in other terrestrial ecosystems. However, to our current knowledge, the patterns and drivers of C/N/P stoichiometry in different organs of riparian plants under diverse inundation scenarios remain poorly documented.

To address this research gap, we gathered plant samples from diverse life forms (annuals and perennials) and organs (leaves, stems, and roots) in the Three Gorges Reservoir Riparian in China. Our objective was to scrutinize the C/N/P stoichiometry across different organs and life forms in the predominant plants of the TGRR while simultaneously identifying the environmental factors associated with these patterns. Specifically, we hypothesized that (1) plants would exhibit distinct C/N/P stoichiometric patterns among organs and life forms due to varying physiological functions and adaptive strategies, and that (2) C/N/P stoichiometry in various organs is strongly influenced by both intrinsic factors (such as life forms) and environmental factors.

## 2 Materials and methods

### 2.1 Study area

**Figure 1:**
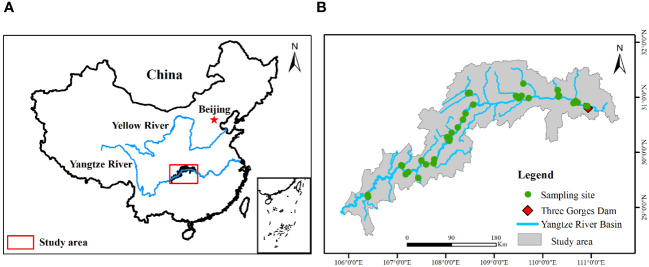
Location of sampling sites **(B)** in the riparian zone of the Three Gorges Reservoir **(A)** in China.

The study area is situated in the upper reaches of the Yangtze River (28°17′~ 32°05′N, 105°73′~ 111°12′E) (**Figure 1**), covering a total area of 344.22 km^2^ and spanning 639.38 km from the Three Gorges Dam to Jiangjin County, Chongqing Municipality (Zheng et al., 2023). This region experiences a humid subtropical monsoon climate, boasting an average annual temperature of 18.22 ± 0.56°C and an average annual precipitation of 1110 ± 75.23 mm. The peak annual flow in the study area predominantly occurs during the summer months (June to September). Chinese soil classification identifies soils in this region as purple and yellow.

Since the operation of the Three Gorges Dam, a distinct riparian zone has emerged, witnessing inundation during the winter months (September – April) instead of conventional summer (May – August) inundation. This alteration, contrary to the natural hydrological cycle, is global and associated with large reservoirs. The water level in this riparian zone fluctuates annually within the elevation range of 145 – 175 m, resulting in a 30 m inundation depth (Arif et al., 2023). During the summer months, when the TGRR is exposed, surrounding vegetation is exposed to high temperatures for one to several weeks and is susceptible to frequent natural flooding, particularly in the area between elevations 140 – 160 m. Consequently, this zone exhibits consistent inundation intensity and frequency due to water level management patterns and the comparable impacts of natural flooding. We categorized the TGRR into four elevation zones based on the duration and depth of inundation (Zheng et al., 2021b), namely 145 – 160 (duration of inundation 230 days, depth of inundation 15 – 30 m), 160 – 165 (duration of inundation 140 days, depth of inundation 10 – 15 m), 165 – 170 (duration of inundation 90 days, depth of inundation 5 – 10 m), and 170 – 175 m (duration of inundation 50 days, depth of inundation 0-5 m).

Due to the inversion of the inundation season and the amplified depth and duration of inundation, the original vegetation of the TGRR has undergone substantial changes. Species like shrubs and trees have disappeared, making way for several perennials with enhanced inundation tolerance, such as *Cynodon dactylon* and *Hemarthria compressa*. These species exhibit robust adaptive capacities in the changing watershed environment, playing a crucial role in sustaining biodiversity and ecosystem functioning in the TGRR (Zheng et al., 2021b). This ecological shift not only altered the vegetation species composition but also introduced 20% of highly competitive annuals (Zhang and Xie, 2021) like *Xanthium strumarium* and *Persicaria lapathifolia*. Flourishing under new riparian zone conditions, these plants form distinct vegetation communities. Consequently, annual and perennial herbaceous plants dominate the entire TGRR, with only a few plant species prevailing, indicating significant community similarity.

### 2.2 Sample collection and measurement

During the peak plant growth period from June to August 2019, an extensive survey and sampling effort of plants and soils was conducted at various elevation zones within the TGRR. A total of 36 representative sample sites were meticulously identified (**Figure 1**), leveraging TGRR jurisdiction staff expertise familiar with the area. This collaboration facilitated the selection of sites with minimal anthropogenic disturbance and allowed us to circumvent the challenges posed by steep slopes and landslides. Each sample site in every elevation zone, spanning 100 m, was designated with the assistance of a 100-m-long survey transect (Zheng et al., 2021b). Notably, at the tail end of the reservoir, sampling was confined to zones 165 – 170 and/or 170 – 175 due to elevated water levels.

Within each transect, three randomly chosen and relatively evenly vegetated quadrats (2 m × 2 m) were identified for assessing species composition, species count, community cover, and height. Community cover denotes the area enveloped by all vegetation in the sample. The average height of all species in the community was measured using a ruler with a scale. Subsequently, a minimum of five individuals of each species in every transect were selected. The complete root system of each target plant was excavated with a spade. The species were then categorized into annuals and perennials based on the Flora of China. Plant samples, including leaves, stems, and roots, were dried at 65°C for 72 hours. Simultaneously, a 500 g surface (0 ~ 20 cm) soil sample was collected from each quadrat. After removing the stones, these samples were naturally dried and screened using a 2 mm filter. Ultimately, 29 dominant species occurring more than three times throughout the sample sites were chosen for subsequent analysis (Yan et al., 2019). These 29 species belong to 10 families and 26 genera (**Supplementary Table S1**), comprising 18 annuals (140 individuals, 420 leaf/stem/root samples) and 11 perennials (125 individuals, 375 leaf/stem/root samples). Thus, 795 samples (265 leaves, 265 stems, and 265 roots) were analyzed in this study.

In the laboratory, dried plants (leaves, stems and roots) and soil samples were finely ground into powder. The C and N contents of the samples were determined using an elemental analyzer (Elementar Vario EL, Germany), and the P contents were determined by ICP-OES (Loughborough, UK). Soil pH was accurately determined by an acidimeter, while the soil water mix was maintained at a ratio of 1:2.5. Soil water content was assessed by drying the soil in an oven at 105°C for 48 h. Soil bulk density is the ratio of the volume of a soil core sample to the mass of dry soil.

### 2.3 Statistical analysis

Differences in C, N, and P concentrations and their stoichiometric ratios among organs (roots, stems and leaves) and between life forms (annual and perennial species) were tested using a nonparametric Kruskal– Wallis test and pairwise Wilcox multiple comparison test. To assess the relative contributions of drivers to the concentrations and ratios of these nutrients, we employed random forest analysis using the *randomForest* package (Luo et al., 2019). The Random Forest algorithm, capable of incorporating numerous predictors, delivers precise predictions and interpretations. The importance of the selected variables to the Random Forest model was quantified using the percentage increase in mean square error.

Subsequently, we examined the direct and indirect effects of drivers influencing changes in C/N/P stoichiometry within various organs. This analysis was based on a hierarchical path of *a priori* knowledge and conceptual models (**Supplementary Table S2**) using segmented structural equation modeling (SEM) (Lefcheck and Freckleton, 2015). Segmented SEM extends traditional SEM by considering the contribution of random variables to response variables. Variables exhibiting non-significant or covariate effects on changes in C/N/P stoichiometry were eliminated before subjecting the data to SEM analysis. To account for random effects in the segmented SEM, a linear mixed model with sample sites as random factors was implemented using the piecewiseSEM software package (Luo et al., 2019). We estimated the overall fit of the model by Fisher’s C-value, and the model was considered to be adequately fitted to the data when the model had a Fisher’s C-value of *P* > 0.05 (Lefcheck and Freckleton, 2015). All the above analyses were conducted using R 4.3.2 (R Core Team, 2023).

## 3 Results

### 3.1 Riparian plant C/N/P concentrations and stoichiometry ratios

**Figure 2:**
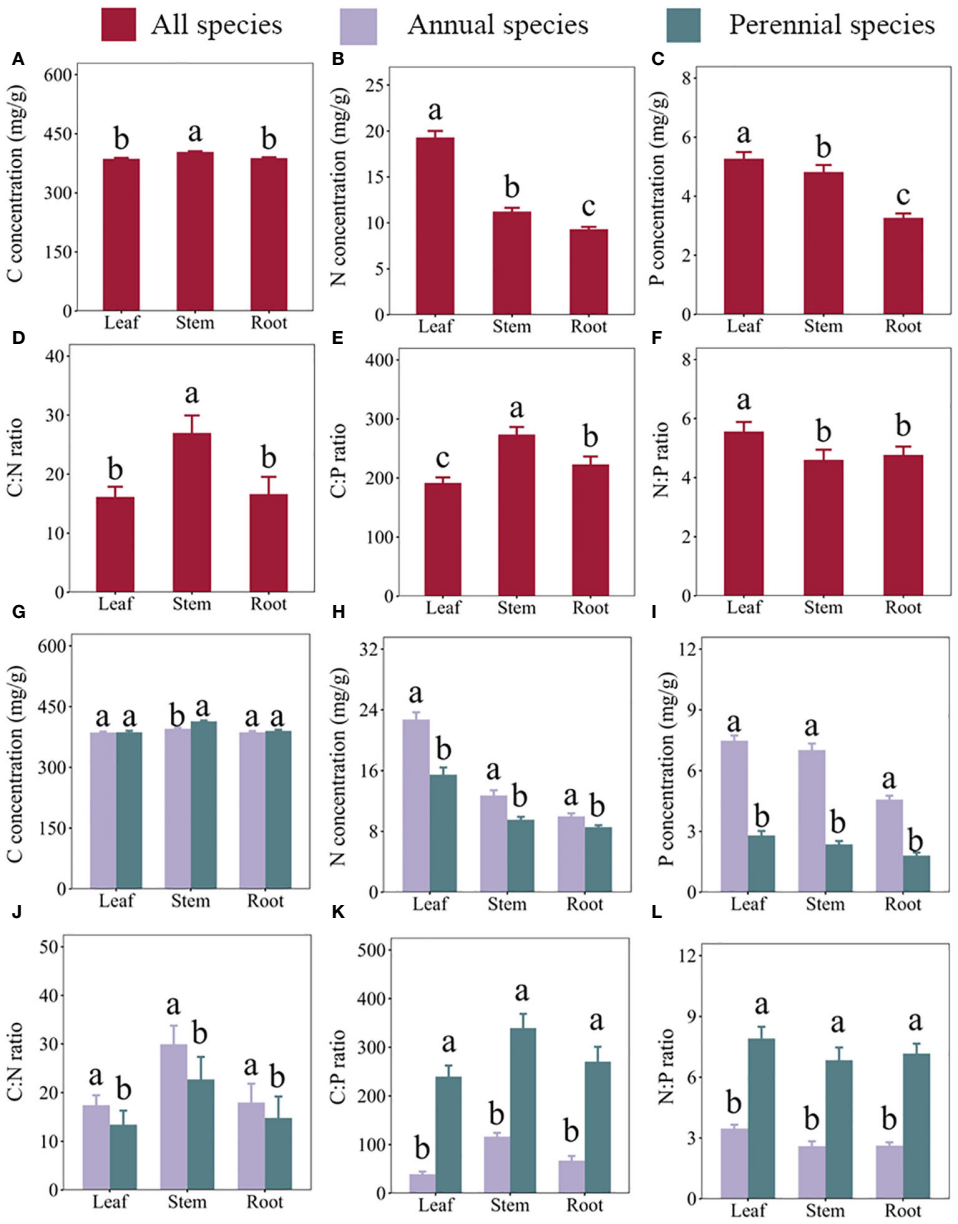
Carbon (C), nitrogen (N), and phosphorus (P) concentrations and their stoichiometric ratios in three organs (leaves, stems, and roots) of plants growing in the riparian zone of the Three Gorges Reservoir in China. **(A–F)** represent C concentrations, N concentrations, P concentrations, C:N ratios, C:P ratios, and N:P ratios in different organs across all species, respectively. **(G–L)** represent C concentrations, N concentrations, P concentrations, C:N ratios, C:P ratios, and N:P ratios, respectively, in the leaves, stems, and roots of annual and perennial plants. Error bars indicate the standard error of the mean. Different lowercase letters denote significant differences among organs.

Our results revealed variations in plant concentrations of C, N, and P, as well as C:N, C:P, and N:P ratios among different organs and across life types within the TGRR ecosystem (refer to **Figure 2**; **Supplementary Table S3**). Specifically, leaf concentrations of C, N, and P were measured at 386.65, 19.31, and 5.27 mg/g, respectively, with corresponding C:N, C:P, and N:P ratios of 16.15, 191.7, and 5.56. Stem concentrations were slightly higher, with values of 404.02, 11.23, and 4.81 mg/g for C, N, and P, accompanied by C:N, C:P, and N:P ratios of 26.98, 273.72, and 4.6. Meanwhile, root concentrations were 388.22, 9.32, and 3.27 mg/g for C, N, and P, with corresponding C:N, C:P, and N:P ratios of 16.63, 223.06, and 4.77.

Comparing the organs, leaf C concentrations (386.65 mg/g) and C:N ratios (16.15) resembled those in roots but were significantly lower than in stems (see **Figures 2A, D**). Leaf N (19.31 mg/g) and P (5.27 mg/g) concentrations, as well as N:P ratios (4.6) were higher (**Figures 2B, C, F**), whereas C:P ratios (191.7) were less than stems and roots (**Figure 2E**). Analyzing annuals and perennials, leaf and root C concentrations exhibited similarity, while the C content in stem (395.29 mg/g) of annual plants was lower than that in stem (413.80 mg g-1) of perennial plants (refer to **Figure 2G**). In contrast to annuals, perennials displayed lower N and P concentrations, as well as lower C:N ratios across all organs (see **Figures 2H–J**). Additionally, perennials exhibited higher C:P and N:P ratios (**Figures 2K–L**) than annuals in all organs.

### 3.2 Riparian plant C/N/P concentrations and stoichiometric ratios in response to inundation

**Figure 3:**
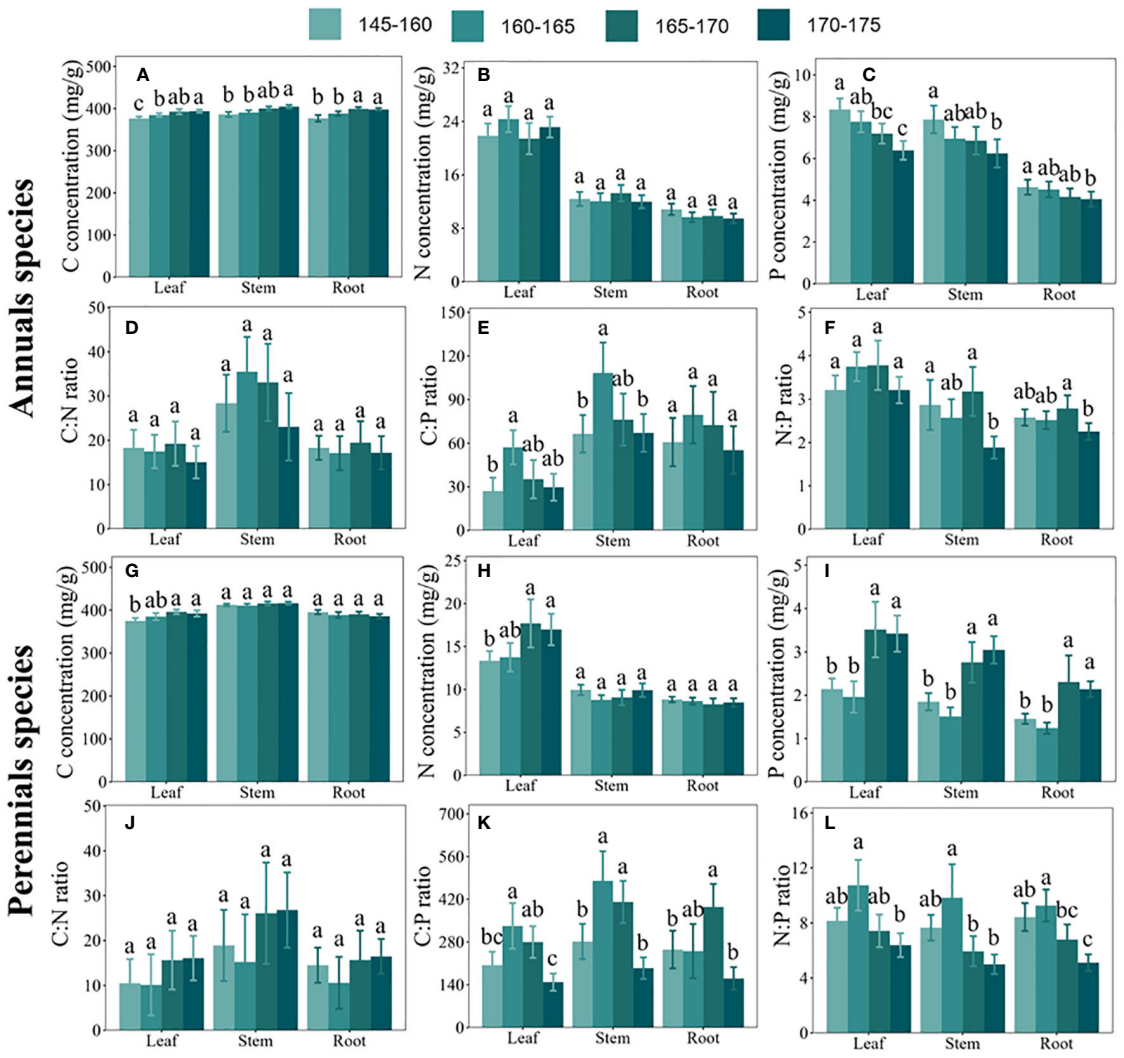
Carbon (C), nitrogen (N), and phosphorus (P) concentrations and their stoichiometric ratios in leaves, stems, and roots of plants growing in different elevation zones. **(A–F)** represent C concentrations, N concentrations, P concentrations, C:N ratios, C:P ratios, and N:P ratios in three organs (leaves, stems, and roots) of annual plants, respectively. **(G–L)** represent C concentrations, N concentrations, P concentrations, C:N ratios, C:P ratios, and N:P ratios in three organs (leaves, stems, and roots) of perennial plants, respectively. Error bars indicate the standard error of the mean. Different lowercase letters denote significant differences among organs.

Our findings indicated distinct responses to C, N, and P concentrations among annual and perennial plants along the inundation gradient. As the elevation increased, C concentrations exhibited a substantial rise in various organs of annuals and leaves of perennials, while no substantial variations were observed in the stems and roots of perennials (see **Figures 3A, G**). Conversely, N concentrations remained relatively stable in different organs of annuals and in the stems and roots of perennials as elevation increased, but showed a significant increase in perennial leaves (see **Figures 3B, H**). Moreover, P concentrations experienced a considerable decrease in various organs of annuals and a significant increase in different organs of perennials with rising elevation (see **Figures 3C, I**).

Furthermore, our analysis revealed that C:N:P stoichiometric ratios in both annuals and perennials exhibited similar responses to the inundation gradient. Consequently, both C:P and N:P ratios in all organs of annual and perennial plants showed an initial increase followed by a decrease with elevation (see **Figures 3E, F, K, L**), while the C:N ratio remained relatively unchanged (see **Figures 3D, J**).

### 3.3 Effects of drivers on riparian plant C/N/P concentrations and stoichiometric ratios

**Figure 4:**
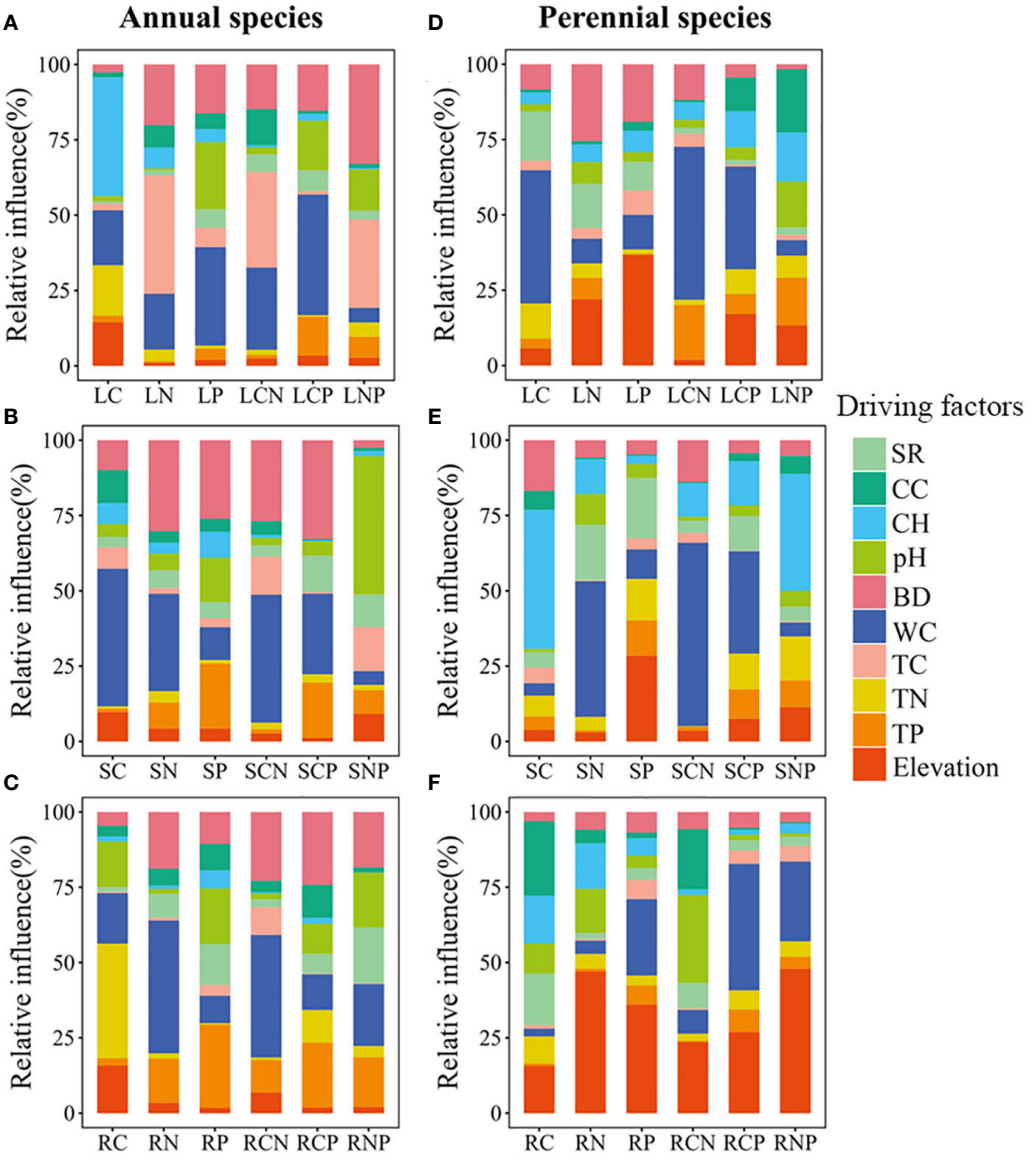
The relative importance of environmental factors on carbon (C), nitrogen (N), phosphorus (P), carbon to nitrogen ratio (CN), carbon to phosphorus ratio (CP), and nitrogen to phosphorus ratio (NP) for different plant organs (leaves, stems, and roots) of annuals **(A–C)** and perennials **(D–F)**. L, S, and R represent leaves, stems, and roots, respectively. SR (species richness), CC (community cover), CH (community height), pH (soil pH values), BD (soil bulk density), WC (soil water content), TC (total soil carbon), TN (total soil nitrogen), TP (total soil phosphorus), and elevation (lower values indicate greater inundation intensity) are used in these panels.

Random forest analyses revealed that variations in C/N/P concentrations and stoichiometric ratios in different organs were predominantly attributed to soil properties rather than plant communities (see **Supplementary Figure S1**). Our results further indicated that the C/N/P concentrations and stoichiometric ratios in various organs across different life types were influenced by distinct factors (refer to **Figure 4**). In annual plants, community height, bulk density, water content, and total carbon emerged as the main factors affecting leaves (see **Figure 4A**). pH values, bulk density, and water content played pivotal roles for stems (see **Figure 4B**), while bulk density, water content, total nitrogen, and total phosphorus were significant factors for roots (see **Figure 4C**). In perennials, community cover, elevation, water content, and bulk density were identified as major factors for leaves (see **Figure 4D**). Water content, elevation, and community height were crucial factors for stems (see **Figure 4E**), and pH values, community cover, elevation, and water content were the primary factors for roots (see **Figure 4F**).

**Figure 5:**
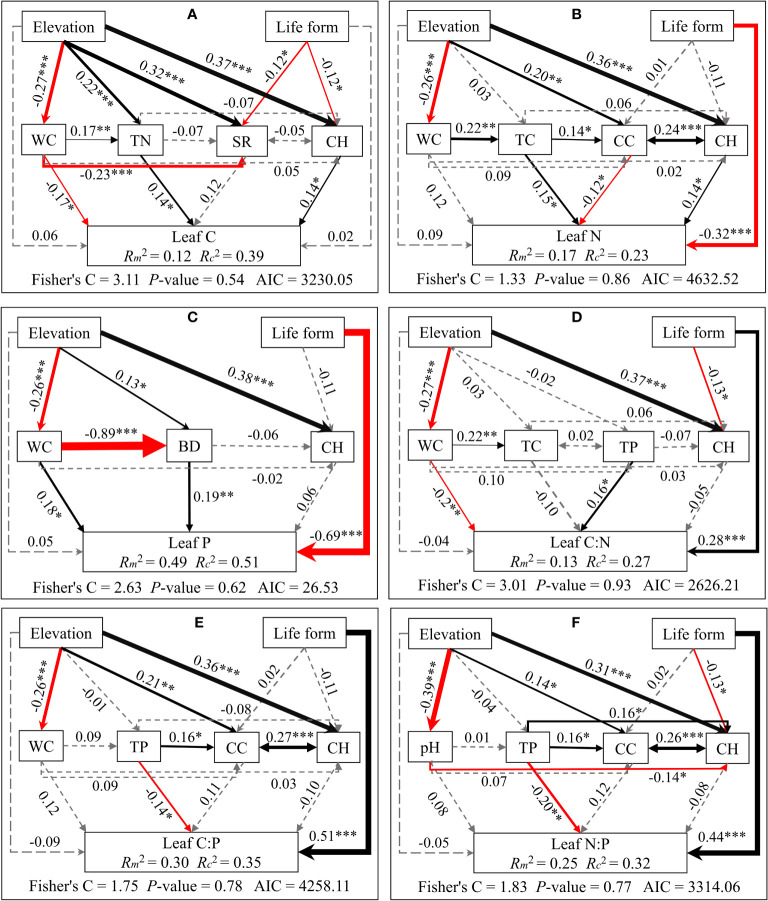
Piecewise structural equation modeling considering the effects of elevation, life forms, plant communities, and soil factors on leaf C **(A)**, N **(B)**, P **(C)**, C:N **(D)**, C:P **(E)**, and N:P **(F)**. Coefficients are standardized predictive coefficients for each causal pathway. Red and black arrows indicate positive and negative relationships, respectively. Dashed arrows indicate non-significant paths (p > 0.05). Arrow thickness is proportional to the absolute value of the standardized coefficient. Marginal R^2^ (R^2^m) and conditional R^2^ (R^2^c) represent the variances explained by fixed effects and fixed and random effects, respectively. Significance levels are denoted as *P < 0.05, **P < 0.01, and ***P < 0.001. Abbreviations have the same meaning as in **Figure 4**.

**Figure 6:**
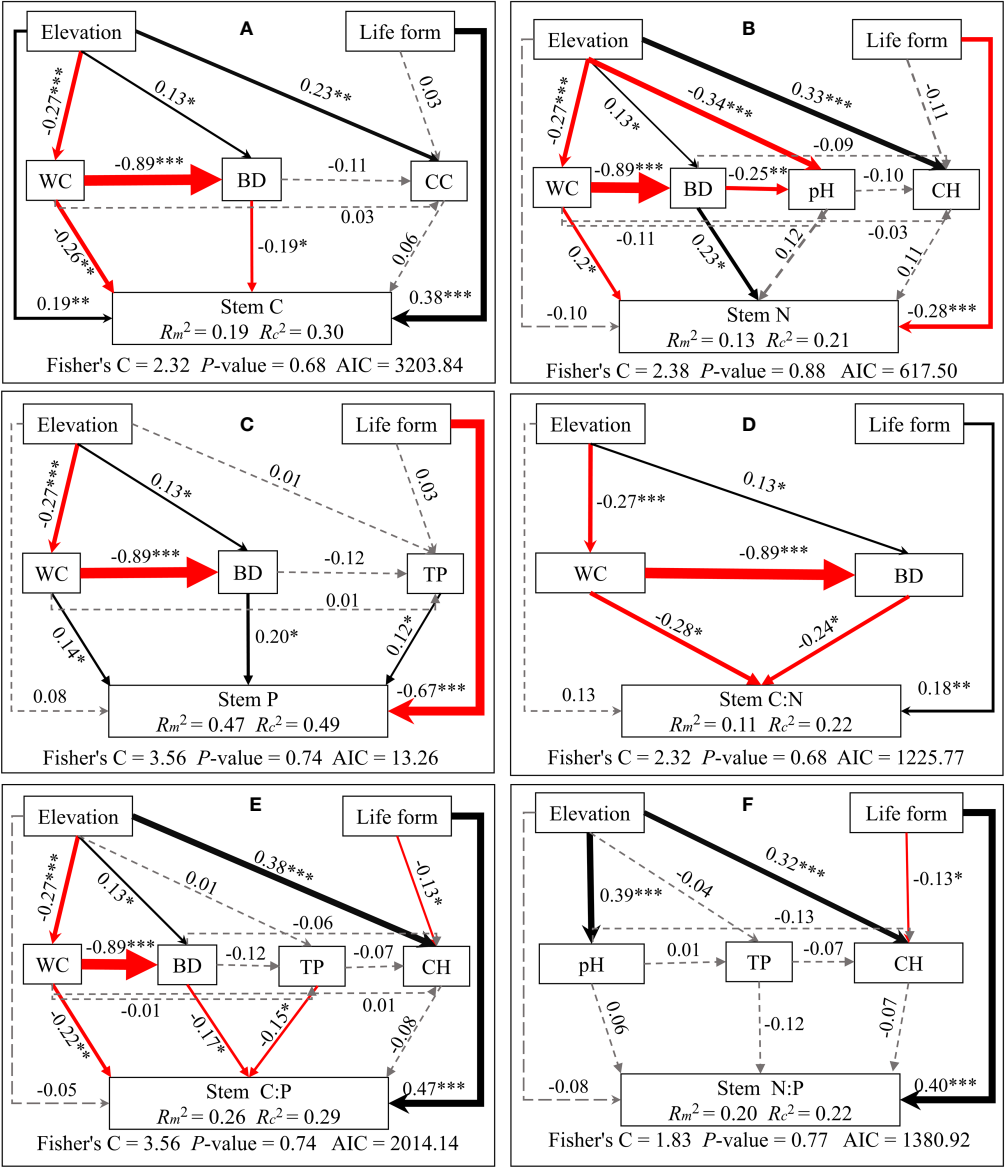
Piecewise structural equation modeling considering the effects of elevation, life forms, plant communities, and soil factors on stem C **(A)**, N **(B)**, P **(C)**, C:N **(D)**, C:P **(E)**, and N:P **(F)**. Coefficients are standardized predictive coefficients for each causal pathway. Red and black arrows indicate positive and negative relationships, respectively. Dashed arrows indicate non-significant paths (p > 0.05). Arrow thickness is proportional to the absolute value of the standardized coefficient. Marginal R^2^ (R^2^m) and conditional R^2^ (R^2^c) represent the variances explained by fixed effects and fixed and random effects, respectively. Significance levels are denoted as *P < 0.05, **P < 0.01, and ***P < 0.001. Abbreviations have the same meaning as in **Figure 4**.

**Figure 7:**
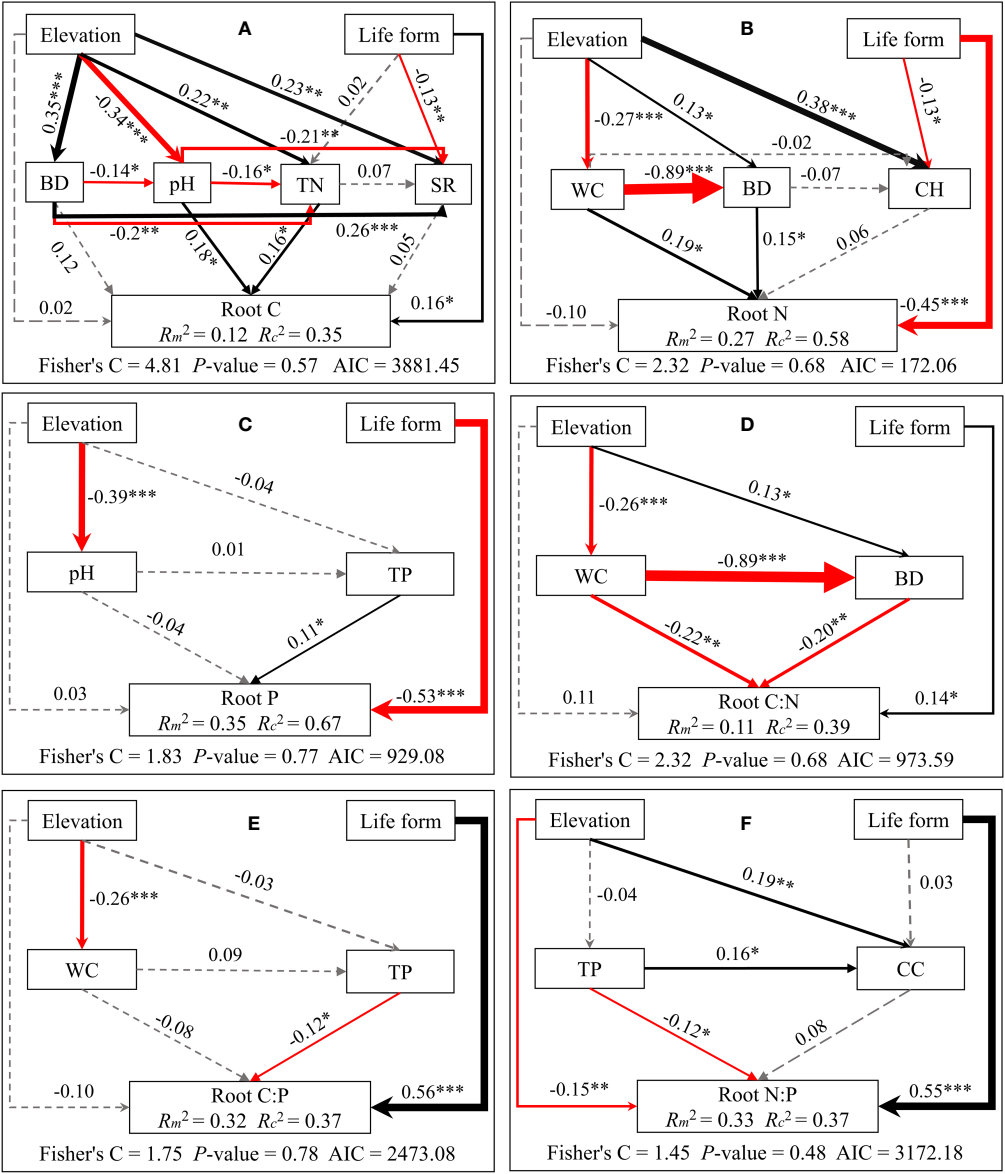
Piecewise structural equation modeling considering the effects of elevation, life forms, plant communities, and soil factors on root C **(A)**, N **(B)**, P **(C)**, C:N **(D)**, C:P **(E)**, and N:P **(F)**. Coefficients are standardized predictive coefficients for each causal pathway. Red and black arrows indicate positive and negative relationships, respectively. Dashed arrows indicate non-significant paths (p > 0.05). Arrow thickness is proportional to the absolute value of the standardized coefficient. Marginal R^2^ (R^2^m) and conditional R^2^ (R^2^c) represent the variances explained by fixed effects and fixed and random effects, respectively. Significance levels are denoted as *P < 0.05, **P < 0.01, and ***P < 0.001. Abbreviations have the same meaning as in **Figure 4**.

Structural equation modeling analyses demonstrated that elevation indirectly influences C/N/P concentrations and stoichiometric ratios of different plant organs. This is mainly through alterations in soil physicochemical properties and plant community properties. Meanwhile, life forms directly impact C/N/P concentrations and stoichiometric ratios (see **Figures 5**–**7**). Plant community properties, such as community height and cover, significantly affected leaf C and N concentrations (see **Figures 5A, B**). Conversely, soil physical and chemical properties had both direct and indirect effects on C/N/P concentrations and stoichiometric ratios in different organs (see **Figures 5**-**7**). Specifically, leaf C, N, and P concentrations were directly influenced by soil total nitrogen, total carbon, and bulk density, respectively, and directly or indirectly affected by water content (see **Figures 5A–C**). Furthermore, leaf C, N, and P stoichiometric ratios were strongly impacted by soil total phosphorus (see **Figures 5D–F**). Soil water content had a direct effect on C, N, and P concentrations and stoichiometric ratios of stems and indirectly influenced these attributes via changes in soil bulk density, whereas soil P had a significant direct effect on P concentrations and C:P ratios of stems (see **Figures 6A–F**). C concentrations in plant roots were directly influenced by pH and total nitrogen (see **Figure 7A**), and root P, C:P, and N:P were directly affected by total phosphorus (see **Figures 7C, E, F**). Soil water content had a direct effect on root N and C:N and indirectly affected these attributes via changes in soil bulk density (see **Figures 7B, D**).

## 4 Discussion

### 4.1 Patterns of different plant life forms and organ elements in riparian ecosystems

Our results revealed that C concentrations in leaves, stems, and roots were significantly lower than those observed in grasslands and forests globally and in China (Ma et al., 2018; Tang et al., 2018), but N and P concentrations were relatively high (see **Supplementary Table S3**), aligning with findings from previous studies in subtropical riparian ecosystems (Yu et al., 2019). Notably, when compared with the Li River riparian zone (Huang et al., 2019), C contents in plant leaves were lower in the TGRR. Besides geographical and climatic factors (Reich and Oleksyn, 2004), the primary explanation for these differences lies in the contrasting seasonal submersion patterns of the two areas, which influence plant carbon sequestration. A recent TGRR study highlighted reduced carbon sequestration due to flooding stress inhibiting photosynthetic efficiency (Ding et al., 2022). Additionally, elements such as iron (Fe) and potassium (K) emerged as limiting factors in plant growth, contributing to low C concentrations in riparian plants (Sardans et al., 2021). Interestingly, N and P contents in the different organs of riparian plants were significantly higher than those observed in grassland and forest ecosystems globally and in China (see **Supplementary Table S3**). One possible explanation is that prolonged inundation in the TGRR accelerates soil mineralization, facilitating N and P uptake by plants (Ye et al., 2020). Furthermore, frequent summer precipitation in the TGRR may transport upland surface pollutants like N and P to the adjacent zone (Zheng et al., 2023), enhancing nutrient recharge for riparian plant growth (Huang et al., 2019). This underscores the conclusion that the newly formed riparian habitat in the TGRR shapes a distinctive ecological strategy for plants. This is characterized by weaker carbon sequestration and stronger nutrient uptake than other terrestrial ecosystems.

The stoichiometry of C, N, and P in plants indicates nutrient utilization efficiency and growth limitations. According to the growth-rate hypothesis (Tian et al., 2018), plants with high growth rates exhibit lower C:N, C:P, and N:P ratios due to increased requirements for N and P during cell proliferation. In our study, the C:N, C:P, and N:P ratios of plant leaves in the TGRR were generally lower than Chinese and global terrestrial plants (see **Supplementary Table S3**), suggesting higher growth rates, lower nutrient-use efficiencies, and reduced carbon assimilation capacity. This aligns with findings from previous studies in natural riparian ecosystems (Huang et al., 2019; Yu et al., 2019) and may be attributed to higher soil P concentrations. The N:P ratio, indicating plant nutrient acquisition and physiological processes, is commonly used to determine N or P limitations (Gusewell, 2004). Our study found N:P ratios (ranging from 4.6 to 5.56) of different organs to be less than 10, indicating N limitation in TGRR plants. This aligns with reports that riparian zones are more susceptible to N limitation than P limitation (Huang et al., 2019; Yu et al., 2019). Periodic inundation of the TGRR results in soil N loss, decreased N mineralization, increased denitrification, and diminished nutrient feedback mechanisms between plants and soil (Ye et al., 2019). Global meta-analysis further suggests that flooding may exacerbate plant N limitation (Cao et al., 2022), though evidence also exists for P limitation in riparian plants (Zhou et al., 2021; Zhang et al., 2021b). The shift from P-limitation to N-limitation in riparian plants might be influenced by higher nutrient inputs from anthropogenic activities (Xia et al., 2014; Ding et al., 2022). However, using N:P ratios to evaluate nutrient limitation has uncertainties, as highlighted by Yan et al. (2017), necessitating consideration of more reliable metrics in future investigations, such as the ratio of leaf N/P uptake efficiency (Du et al., 2020).

Moreover, our findings indicated that plants allocate more N and P to leaves than to stems and roots (see **Figure 2**), revealing distinct nutrient acquisition strategies in riparian habitats. This allocation aligns with the growth rate hypothesis, where leaves, with their rapid metabolism and growth rates, accumulate more nutrients to support carbon sequestration through photosynthesis (Tian et al., 2018). Previous studies have shown that plants will allocate more N and P elements to leaves for photosynthesis and transpiration, particularly when N and P elements are limited, which explains why the N and P concentrations in leaves are higher than in other organs (Xing et al., 2022). In contrast, stems and roots, primarily responsible for water and nutrient absorption and delivery to leaves, exhibit lower concentrations of N and P (Luo et al., 2021). Stems, with their supportive role, require higher structural elements like C for plant morphological construction, explaining the high C concentrations, C:N, and C:P ratios found in stems, as reported in the TGRR (Ding et al., 2022). The higher N:P ratio in leaves may reflect the plant’s ability to mitigate leaf N limitation more effectively in the context of overall N limitation, consistent with plant adaptation strategies observed in other riparian habitats (Zhang et al., 2019). Additionally, our study reveals that annual herbaceous plants exhibit higher N and P concentrations in different organs than perennial plants (see **Figure 2**). This supports the general concept that short-lived, fast-growing species tend to have higher N and P contents than long-lived, slow-growing species (Han et al., 2011; Sardans et al., 2021). Specifically, C and N concentrations were higher in annual plants, indicating increased N utilization efficiency (Ding et al., 2022). Higher C:P and N:P ratios in perennial leaves, stems, and roots compared to annuals support the idea that resistant plants tend to have higher N:P ratios (Sardans et al., 2021). The observed increase in P limitation with rising N:P ratios and the greater sensitivity of perennials to P limitation, as opposed to annuals being more sensitive to N limitation in the TGRR, provide compelling evidence that, in dynamic riparian habitats, plants exhibit diverse elemental stoichiometric patterns among different organs and life forms, confirming our initial hypothesis.

### 4.2 Effects of inundation on different life forms and organ elements of riparian plants

Our findings showed that N and P concentrations in different life forms respond differentially to inundation stress. The results reveal a decrease in P concentration in annual plants as elevation increases, contrasting with the increase in N and P concentrations in perennial plants (see **Figure 3**). This divergence can be attributed to variations in photosynthetic capacities, life cycles, and root structures among different life forms. For instance, competitive annual herbaceous plants in the TGRR habitats, functioning as opportunists and thriving under heightened flooding (Su et al., 2020), exhibit elevated N or P concentrations, corresponding to heightened growth rates and relatively low C:N and C:P ratios (Huang et al., 2019). Notably, a robust positive correlation exists between leaf N concentration and the light saturation rate of net photosynthesis CO_2_ assimilation. Moreover, P, a vital macronutrient essential for photosynthesis, directly participates in photophosphorylation and carbon assimilation (Ding et al., 2022). Therefore, plants in severely inundated areas respond to inundation by enhancing the relative nutrient levels in their leaves, thereby boosting their photosynthetic capacity to rapidly synthesize additional photosynthetic storage products (Huang et al., 2019). Additionally, perennial plants tend to store more nutrients in their roots, stems, and leaves, boasting intricate deep root structures to regenerate after flooding (Zheng et al., 2021b). In contrast, annual plants develop shallower root systems to swiftly absorb water and nutrients during flooding, relying primarily on seeds for survival (Zheng et al., 2021b; Poppenwimer et al., 2023).

Furthermore, our examination of C:N:P stoichiometry across different life forms revealed similar responses to flooding stress. Specifically, the C:P and N:P ratios exhibited an initial increase followed by a decrease on elevated terrain, while C:N remained relatively stable (see **Figure 3**). A plausible explanation for the maintenance of comparable stoichiometric ratios in response to flood stress across diverse plant life forms lies in an adaptive strategy adopted by plants to cope with comparable resource utilization pressures within a given ecosystem (Zheng et al., 2021b; Ding et al., 2022). Prior studies have posited that similar hydrologic stress in the TGRR may amplify the similarity in nutrient resources required by plants at varying elevations (Zheng et al., 2021a). Additionally, characteristics of the plant community, such as community biomass, total cover, and species diversity, displayed a pattern of increasing and then decreasing with rising elevation under the influence of hydrologic disturbance in the TGRR (Chen et al., 2020). This observation partly elucidates the trend in plant C:P and N:P ratios with elevation in the TGRR, given the close association between plant biomass accumulation and C and P concentrations (Xiong et al., 2021). Our findings contribute significantly to understanding plants’ ecological responses to environmental changes and their implications for ecosystem stability and function.

### 4.3 Factors influencing different organ elements in riparian plants under inundation intensity

Previous research has demonstrated that alterations in vegetation community composition and soil nutrients play a pivotal role in shaping C/N/P stoichiometry in plants (Wang et al., 2020; Ding et al., 2022). This process is further modulated by flooding intensity (Cao et al., 2022). Considering this, we endeavored to quantify the relative importance of drivers such as plant community and soil factors on the C/N/P stoichiometry among various plant organs, elucidating the cause-and-effect relationships of these factors in influencing nutrient stoichiometry. Our results underscore that the variability in C/N/P stoichiometry in different organs of riparian plants is primarily attributed to soil properties and life forms rather than inundation and plant community characteristics. This aligns with recent findings (He et al., 2015a; Luo et al., 2021) emphasizing the dominance of plant life forms and soil nutrient conditions over climatic conditions in determining elemental concentrations and stoichiometry in different plant organs. Furthermore, as the most direct source of plant growth and nutrients, soil properties are largely responsible for elemental concentrations and stoichiometric ratios in plants growing in riparian zones (Ding et al., 2022). From an ecological and evolutionary standpoint, the distinct ecological niches occupied by plants of different life types contribute to variations in their nutrient demands and utilization strategies, providing a more direct reflection of nutrient allocation and utilization strategies (Tian et al., 2018; Hu et al., 2021; Xiong et al., 2021). Recent studies further indicate that riparian plants in the TGRR develop species-specific traits and functional groups following prolonged inundation (Su et al., 2020; Zhang and Xie, 2021). Consequently, our study substantiates that soil properties and life forms are pivotal determinants of C, N, and P stoichiometry in riparian vegetation organs. This aligns with our second hypothesis.

Furthermore, our results reveal that plant life forms and soil properties directly influence the concentrations of C, N, and P in different plant organs. Annual plants appear more focused on rapid growth and reproduction, while perennial plants prioritize long-term nutrition accumulation and storage (Poppenwimer et al., 2023), resulting in divergent elemental content and stoichiometric ratios. Soil factors, including water content, bulk density, and pH, directly or indirectly impact plant growth and nutrient status. This is done by influencing root development, nutrient uptake, and physiological metabolic processes. This underscores the pivotal role of soil moisture in overall nutrient uptake and partitioning in plants (Zhang et al., 2021a; Ding et al., 2022). In addition, the results of the present study showed that soil C, N, and P contents have significant positive effects on the elemental concentrations of each plant organ (see **Figures 5**–**7**), aligning with the “biogeochemical hypothesis” (Reich and Oleksyn, 2004), which suggests a positive correlation between nutrients in the habitat and nutrient concentrations in plants. Our findings are consistent with a recent study in the riparian zone of the TGRR that found a significant positive correlation between C, N, and P concentrations in plant roots, stems, and leaves and C, N, and P concentrations in soil. Additionally, we observe that the height and cover of the plant community significantly influence leaf C and N concentrations, highlighting the role of community characteristics in shaping leaf growth and stoichiometric traits by impacting factors like light access and competitive interactions among plants (Ning et al., 2021; Zhang et al., 2021a). This underscores the competitive and synergistic relationships among plants within the community. Furthermore, our study suggests that flooding can directly impact N concentrations in stems and N:P ratios in roots. This can alter elemental concentrations and stoichiometric ratios in various plant organs by modifying soil physicochemical characteristics and community height. In conclusion, this research affirms the significance of inundation in nutrient cycling in riparian plant-soil systems and emphasizes the intricate and diverse influences in riparian habitats affecting plant elemental concentrations and stoichiometric ratios.

However, several limitations should be considered. Firstly, while our study focused on total nutrients rather than available N and P, total nutrients remain a valid indicator of soil nutrient levels. Secondly, our study encompassed an array of environmental factors but omitted climatic factors like average annual precipitation and temperature. This omission is attributed to the relatively narrow range of mean annual precipitation and temperature in our study area, where climatic factors influencing plant elemental stoichiometry typically operate on regional or global scales with pronounced climatic gradients (Han et al., 2011). Lastly, due to sampling difficulties and high labor costs, we conducted only one field survey and sample. Hence, continuous monitoring of C, N, and P interactions in riparian plant-soil systems is imperative in the future to enhance our understanding of biogeochemical processes and ecosystem functions in wetlands.

## 5 Conclusions

We summarize this study by examining the variation in C, N, and P stoichiometry across different organs (leaves, stems, and roots) and life forms (annuals and perennials) of dominant plants, alongside their responses to inundation, plant communities, and soil factors in the TGRR region—a novel riparian ecosystem in China. Our results show that riparian plants have higher nitrogen and phosphorus concentrations but lower carbon concentrations and elemental stoichiometry than grasses and forests, indicating that plant growth in the recently established riparian habitats of the TGRR is nitrogen-limited, prompting the development of plant-specific ecological strategies such as diminished carbon sequestration, lower nutrient-use efficiency, and heightened nutrient uptake capacity. Consequently, distinct physiological functions and adaptive strategies result in variations in C/N/P stoichiometry between life forms and organs. For instance, N and P concentrations were higher in leaves compared to stems and roots, and annuals exhibited higher concentrations than perennials. While C/N/P concentrations in different organs of annual and perennial plants exhibited divergent responses to inundation stress, they maintained similar stoichiometric ratios along the inundation gradient. Importantly, the study underscores that variations in C/N/P stoichiometry in different plant organs are more closely tied to soil characteristics than to plant community structure. Unlike direct impacts on life forms, inundation induces indirect changes in plant C/N/P stoichiometry by altering plant community properties and soil factors. This investigation emphasizes that hydrological changes can influence plant community composition and soil nutrient dynamics, impact plant \growth and stoichiometric properties, and ultimately alter plant ecological strategies and biogeochemical cycling in riparian ecosystems. This research is expected to guide the management and conservation of riparian natural communities for enhanced ecological functions and to support future vegetation restoration.

## Data availability statement

The original contributions presented in the study are included in the article/**Supplementary Material**. Further inquiries can be directed to the corresponding authors.

## Author contributions

LW: Conceptualization, Investigation, Methodology, Software, Visualization, Writing – review & editing. MA: Formal analysis, Investigation, Writing – review & editing. JZ: Formal analysis, Investigation, Methodology, Software, Visualization, Writing – review & editing. CL: Funding acquisition, Project administration, Supervision, Writing – review & editing.

## References

[B1] Arif M. Jie Z. Behzad H. M. Changxiao L. (2023). Assessing the impacts of ecotourism activities on riparian health indicators along the Three Gorges Reservoir in China. Ecol. Indic. 155, 111065. doi: 10.1016/j.ecolind.2023.111065

[B2] Cao Y. Tong R. Tan Q. Mo S. Ma C. Chen G. (2022). Flooding influences on the C, N, and P stoichiometry in terrestrial ecosystems: A meta-analysis. Catena. 215, 106287. doi: 10.1016/j.catena.2022.106287

[B3] Chang Y. Zhong Q. Yang H. Xu C. Hua W. Li B. (2022). Patterns and driving factors of leaf C, N, and P stoichiometry in two forest types with different stand ages in a mid-subtropical zone. For. Ecosyst. 9, 100005. doi: 10.1016/j.fecs.2022.100005

[B4] Chen Z. Yuan X. Roß-Nickoll M. Hollert H. Schäffer A. (2020). Moderate inundation stimulates plant community assembly in the drawdown zone of China’s Three Gorges Reservoir. Environ. Sci. Eur. 32, 1–11. doi: 10.1186/s12302-020-00355-0

[B5] Ding D. Arif M. Liu M. Li J. Hu X. Geng Q.. (2022). Plant-soil interactions and C:N:P stoichiometric homeostasis of plant organs in riparian plantation. Front. Plant Sci. 13. doi: 10.3389/fpls.2022.979023 PMC9376457 35979078

[B6] Du E. Terrer C. Pellegrini A. F. A. Ahlström A. Lissa C. Zhao X.. (2020). Global patterns of terrestrial nitrogen and phosphorus limitation. Nat. Geosci. 13, 221–226. doi: 10.1038/s41561-019-0530-4

[B7] Elser J. J. Sterner R. W. Gorokhova E. Fagan W. F. Markow T. A. Cotner J. B.. (2008). Biological stoichiometry from genes to ecosystems. Ecol. Lett. 3, 540–550. doi: 10.1111/j.1461-0248.2000.00185.x

[B8] Grill G. Lehner B. Thieme M. Geenen B. Tickner D. Antonelli F.. (2019). Mapping the world’s free-flowing rivers. Nature. 569, 215–221. doi: 10.1038/s41586-019-1111-9 31068722

[B9] Gusewell S. (2004). N:P ratios in terrestrial plants: variation and functional significance. New Phytol. 164, 243–266. doi: 10.1111/j.1469-8137.2004.01192.x 33873556

[B10] Han W. Fang J. Guo D. Zhang Y. (2005). Leaf nitrogen and phosphorus stoichiometry across 753 terrestrial plant species in China. New Phytol. 168, 377–385. doi: 10.1111/j.1469-8137.2005.01530.x 16219077

[B11] Han W. X. Fang J. Y. Reich P. B. Ian Woodward F. Wang Z. H. (2011). Biogeography and variability of eleven mineral elements in plant leaves across gradients of climate, soil and plant functional type in China. Ecol. Lett. 14, 788–796. doi: 10.1111/j.1461-0248.2011.01641.x 21692962

[B12] He M. Dijkstra F. A. Zhang K. Tan H. Zhao Y. Li X. (2015a). Influence of life form, taxonomy, climate, and soil properties on shoot and root concentrations of 11 elements in herbaceous plants in a temperate desert. Plant Soil. 398, 339–350. doi: 10.1007/s11104-015-2669-0

[B13] He M. Zhang K. Tan H. Hu R. Su J. Wang J.. (2015b). Nutrient levels within leaves, stems, and roots of the xeric species *Reaumuria soongorica* in relation to geographical, climatic, and soil conditions. Ecol. Evol. 5, 1494–1503. doi: 10.1002/ece3.1441 25897388 PMC4395178

[B14] Hu J. Yu H. Li Y. Wang J. Lv T. Liu C.. (2021). Variation in resource allocation strategies and environmental driving factors for different life-forms of aquatic plants in cold temperate zones. J. Ecol. 109, 1–14. doi: 10.1111/1365-2745.13719

[B15] Huang D. Wang D. Ren Y. (2019). Using leaf nutrient stoichiometry as an indicator of flood tolerance and eutrophication in the riparian zone of the Lijang River. Ecol. Indic. 98, 821–829. doi: 10.1016/j.ecolind.2018.11.064

[B16] Jing X. Su W. Fan S. Luo H. Chu H. (2022). Ecological strategy of *phyllostachys heteroclada oliver* in the riparian zone based on ecological stoichiometry. Front. Plant Sci. 13. doi: 10.3389/fpls.2022.974124 PMC9659970 36388549

[B17] Lefcheck J. S. Freckleton R. (2015). piecewiseSEM: Piecewise structural equation modeling in R for ecology, evolution, and systematics. Methods Ecol. Evol. 7, 573–579. doi: 10.1111/2041-210x.12512

[B18] Li X. Ding C. Bu H. Han L. Ma P. Su D. (2019). Effects of submergence frequency on soil C:N:P ecological stoichiometry in riparian zones of Hulunbuir steppe. J. Soil. Sediment. 20, 1480–1493. doi: 10.1007/s11368-019-02533-x

[B19] Lozanovska I. Rivaes R. Vieira C. Ferreira M. T. Aguiar F. C. (2020). Streamflow regulation effects in the Mediterranean rivers: How far and to what extent are aquatic and riparian communities affected? Sci. Total Environ. 749, 141616. doi: 10.1016/j.scitotenv.2020.141616 32827828

[B20] Lu J. Zhao X. Wang S. Feng S. Ning Z. Wang R.. (2023). Untangling the influence of abiotic and biotic factors on leaf C, N, and P stoichiometry along a desert-grassland transition zone in northern China. Sci. Total Environ. 884, 163902. doi: 10.1016/j.scitotenv.2023.163902 37137371

[B21] Luo Y. H. Cadotte M. W. Burgess K. S. Liu J. Tan S. L. Zou J. Y.. (2019). Greater than the sum of the parts: How the species composition in different forest strata influence ecosystem function. Ecol. Lett. 22, 1449–1461. doi: 10.1111/ele.13330 31267650

[B22] Luo Y. Peng Q. Li K. Gong Y. Liu Y. Han W. (2021). Patterns of nitrogen and phosphorus stoichiometry among leaf, stem and root of desert plants and responses to climate and soil factors in Xinjiang, China. Catena. 199, 105100. doi: 10.1016/j.catena.2020.105100

[B23] Ma S. He F. Tian D. Zou D. Yan Z. Yang Y.. (2018). Variations and determinants of carbon content in plants: A global synthesis. Biogeosciences. 15, 693–702. doi: 10.5194/bg-15-693-2018

[B24] Ning Z. Zhao X. Li Y. Wang L. Lian J. Yang H.. (2021). Plant community C:N:P stoichiometry is mediated by soil nutrients and plant functional groups during grassland desertification. Ecol. Eng. 162, 106179. doi: 10.1016/j.ecoleng.2021.106179

[B25] Poppenwimer T. Mayrose I. DeMalach N. (2023). Revising the global biogeography of annual and perennial plants. Nature. 624, 1–6. doi: 10.1038/s41586-023-06644-x PMC10830411 37938778

[B26] R Core Team (2023). R: A language and environment for statistical computing (Vienna, Austria: R Foundation for Statistical Computing). Available at: https://www.R-project.org/.

[B27] Reich P. B. Oleksyn J. (2004). Global patterns of plant leaf N and P in relation to temperature and latitude. Proc. Natl. Acad. Sci. U.S.A. 101, 11001–11006. doi: 10.1073/pnas.0403588101 15213326 PMC503733

[B28] Sardans J. Janssens I. A. Ciais P. Obersteiner M. Peñuelas J. (2021). Recent advances and future research in ecological stoichiometry. Perspect. Plant Ecol. 50, 125611. doi: 10.1016/j.ppees.2021.125611

[B29] Sardans J. Rivas-Ubach A. Peñuelas J. (2012). The C:N:P stoichiometry of organisms and ecosystems in a changing world: A review and perspectives. Perspect. Plant Ecol. 14, 33–47. doi: 10.1016/j.ppees.2011.08.002

[B30] Spohn M. Bagchi S. Biederman L. A. Borer E. T. Brathen K. A. Bugalho M. N.. (2023). The positive effect of plant diversity on soil carbon depends on climate. Nat. Commun. 14, 6624. doi: 10.1038/s41467-023-42340-0 37857640 PMC10587103

[B31] Su X. L. Bejarano M. D. Yi X. M. Lin F. Ayi Q. Zeng B. (2020). Unnatural flooding alters the functional diversity of riparian vegetation of the Three Gorges Reservoir. Freshw. Biol. 65, 1585–1595. doi: 10.1111/fwb.13523

[B32] Tang Z. Xu W. Zhou G. Bai Y. Li J. Tang X.. (2018). Patterns of plant carbon, nitrogen, and phosphorus concentration in relation to productivity in China's terrestrial ecosystems. Proc. Natl. Acad. Sci. U. S. A. 115, 4033–4038. doi: 10.1073/pnas.1700295114 29666316 PMC5910803

[B33] Tian D. Yan Z. Niklas K. J. Han W. Kattge J. Reich P. B.. (2018). Global leaf nitrogen and phosphorus stoichiometry and their scaling exponent. Natl. Sci. Rev. 5, 728–739. doi: 10.1093/nsr/nwx142

[B34] Wang M. Gong Y. Lafleur P. Wu Y. (2021). Patterns and drivers of carbon, nitrogen and phosphorus stoichiometry in Southern China's grasslands. Sci. Total Environ. 785, 147201. doi: 10.1016/j.scitotenv.2021.147201

[B35] Wang Y. Ren Z. Ma P. Wang Z. Niu D. Fu H.. (2020). Effects of grassland degradation on ecological stoichiometry of soil ecosystems on the Qinghai-Tibet Plateau. Sci. Total Environ. 722, 137910. doi: 10.1016/j.scitotenv.2020.137910 32192971

[B36] Xia C. Yu D. Wang Z. Xie D. (2014). Stoichiometry patterns of leaf carbon, nitrogen and phosphorous in aquatic macrophytes in eastern China. Ecol. Eng. 70, 406–413. doi: 10.1016/j.ecoleng.2014.06.018

[B37] Xing S. Cheng X. Kang F. Wang J. Yan J. Han H. (2022). The patterns of N/P/K stoichiometry in the *Quercus wutaishanica* community among different life forms and organs and their responses to environmental factors in northern China. Ecol. Indic. 137, 108783. doi: 10.1016/j.ecolind.2022.108783

[B38] Xiong J. Dong L. Lu J. Hu W. Gong H. Xie S.. (2021). Variation in plant carbon, nitrogen and phosphorus contents across the drylands of China. Funct. Ecol. 36, 174–186. doi: 10.1111/1365-2435.13937

[B39] Yan Y. Liu Q. Zhang Q. Ding Y. Li Y. (2019). Adaptation of dominant species to drought in the Inner Mongolia Grassland: Species level and functional type level analysis. Front. Plant Sci. 10. doi: 10.3389/fpls.2019.00231 PMC6477032 31040855

[B40] Yan Z. Tian D. Han W. Tang Z. Fang J. (2017). An assessment on the uncertainty of the nitrogen to phosphorus ratio as a threshold for nutrient limitation in plants. Ann. Bot-London. 120, 937–942. doi: 10.1093/aob/mcx106 PMC5710604 29028870

[B41] Ye C. Butler O. M. Chen C. R. Liu W. Z. Du M. Zhang Q. F. (2020). Shifts in characteristics of the plant-soil system associated with flooding and revegetation in the riparian zone of Three Gorges Reservoir, China. Geoderma. 361, 114015. doi: 10.1016/j.geoderma.2019.114015

[B42] Ye C. Chen C. Butler O. M. Rashti M. R. Esfandbod M. Du M.. (2019). Spatial and temporal dynamics of nutrients in riparian soils after nine years of operation of the Three Gorges Reservoir, China. Sci. Total Environ. 664, 841–850. doi: 10.1016/j.scitotenv.2019.02.036 30769308

[B43] Yin H. Zheng H. Zhang B. Tariq A. Lv G. Zeng F.. (2021). Stoichiometry of C:N:P in the roots of *Alhagi sparsifolia* is more sensitive to soil nutrients than aboveground organs. Front. Plant Sci. 12. doi: 10.3389/fpls.2021.698961 PMC8545904 34712247

[B44] Yu M. Tao Y. Liu W. Xing W. Liu G. Wang L.. (2019). C, N, and P stoichiometry and their interaction with different plant communities and soils in subtropical riparian wetlands. Environ. Sci. pollut. R. 27, 1024–1034. doi: 10.1007/s11356-019-07004-x 31820250

[B45] Yu R. P. Zhang W. P. Fornara D. A. Li L. Wainwright C. (2021). Contrasting responses of nitrogen:phosphorus stoichiometry in plants and soils under grazing: A global meta-analysis. J. Appl. Ecol. 58, 964–975. doi: 10.1111/1365-2664.13808

[B46] Zhang J. He N. Liu C. Xu L. Yu Q. Yu G. (2018). Allocation strategies for nitrogen and phosphorus in forest plants. Oikos. 127, 1506–1514. doi: 10.1111/oik.05517

[B47] Zhang A. Li X. Wu S. Li L. Jiang Y. Wang R.. (2021a). Spatial pattern of C:N:P stoichiometry characteristics of alpine grassland in the Altunshan Nature Reserve at North Qinghai-Tibet Plateau. Catena. 207, 105691. doi: 10.1016/j.catena.2021.105691

[B48] Zhang D. Qi Q. Tong S. Wang J. Zhang M. Zhu G.. (2021b). Effect of hydrological fluctuation on nutrient stoichiometry and trade-offs of *Carex schmidtii* . Ecol. Indic. 120, 106924. doi: 10.1016/j.ecolind.2020.106924

[B49] Zhang A. Xie Z. (2021). C_4_ herbs dominate the reservoir flood area of the Three Gorges Reservoir. Sci. Total Environ. 755, 142479. doi: 10.1016/j.scitotenv.2020.142479 33035969

[B50] Zhang J. Zhao N. Liu C. Yang H. Li M. Yu G.. (2017). C:N:P stoichiometry in China's forests: From organs to ecosystems. Funct. Ecol. 32, 50–60. doi: 10.1111/1365-2435.12979

[B51] Zhang X. Zhou J. Guan T. Cai W. Jiang L. Lai L.. (2019). Spatial variation in leaf nutrient traits of dominant desert riparian plant species in an arid inland river basin of China. Ecol. Evol. 9, 1523–1531. doi: 10.1002/ece3.4877 30805179 PMC6374681

[B52] Zheng J. Arif M. He X. Liu X. Li C. (2023). Distinguishing the mechanisms driving multifaceted plant diversity in subtropical reservoir riparian zones. Front. Plant Sci. 14. doi: 10.3389/fpls.2023.1138368 PMC9998900 36909398

[B53] Zheng J. Arif M. Zhang S. Yuan Z. Zhang L. Dong Z.. (2021a). The convergence of species composition along the drawdown zone of the Three Gorges Dam Reservoir, China: implications for restoration. Environ. Sci. pollut. R. 28, 42609–42621. doi: 10.1007/s11356-021-13774-0 33818726

[B54] Zheng J. Arif M. Zhang S. Yuan Z. Zhang L. Li J.. (2021b). Dam inundation simplifies the plant community composition. Sci. Total Environ. 801, 149827–149839. doi: 10.1016/j.scitotenv.2021.149827 34467924

[B55] Zhou Y. Jiao L. Qin H. Li F. (2021). Effect of environmental stress on the nutrient stoichiometry of the clonal Plant phragmites australis in inland riparian wetlands of Northwest China. Front. Plant Sci. 12. doi: 10.3389/fpls.2021.705319 PMC8416684 34490007

