# Supplementary material for: Patterns and drivers of plant carbon, nitrogen, and phosphorus stoichiometry in a novel riparian ecosystem
Source: Front Plant Sci. 2024 Apr 8;15:1354222. doi: 10.3389/fpls.2024.1354222 (PMC11036011; doi:10.3389/fpls.2024.1354222)
Supplement: Supplementary file 1 [file DataSheet_1.docx]

Supplementary Material

Patterns and Drivers of Plant Carbon, Nitrogen, and Phosphorus Stoichiometry in a Novel Riparian Ecosystem

Lei Wang ^1, †^, Muhammad Arif ^1, 2, †^, Jie Zheng ^1, 2,^ *, Changxiao Li ^1, 2,^ *

^1^Key Laboratory of Eco-environments in the Three Gorges Reservoir Region (Ministry of Education), Chongqing Key Laboratory of Plant Ecology and Resources Research in the Three Gorges Reservoir Region, School of Life Sciences, Southwest University, Chongqing 400715, China

^2^Biological Science Research Center, Academy for Advanced Interdisciplinary Studies, Southwest University, Chongqing 400715, China

† These authors contributed equally to this work.

*** Correspondence:**

Jie Zheng (jiezheng@email.swu.edu.cn)

Changxiao Li ([lichangx@swu.edu.cn](mailto:lichangx@swu.edu.cn))

# Supplementary Figures and Tables

## Supplementary Figures


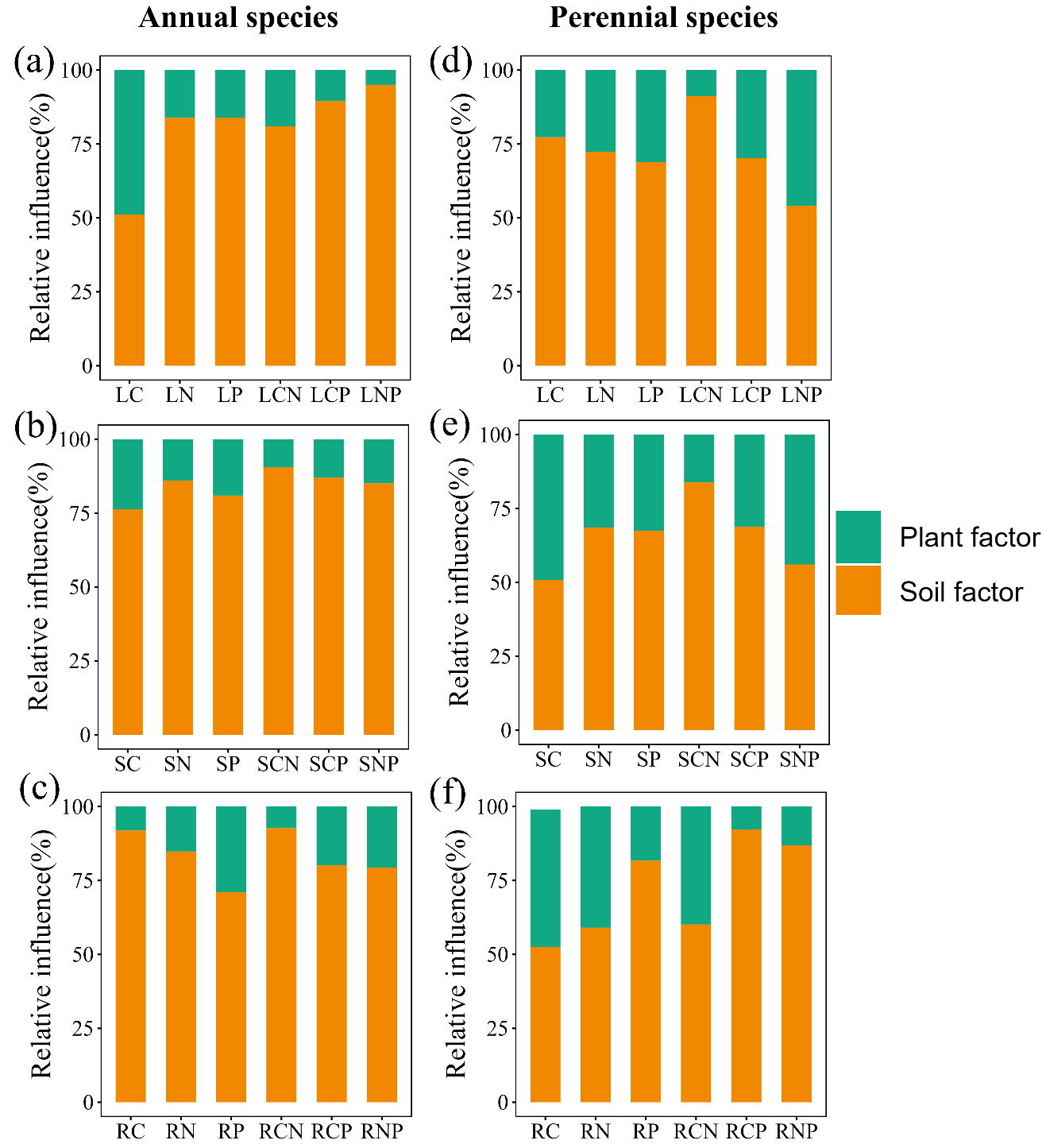


**Figure S1** Relative importance of plant community characteristics and soil factors for carbon (C), nitrogen (N), phosphorus (P), carbon to nitrogen (CN) ratio, carbon to phosphorus (CP) ratio, and nitrogen to phosphorus (NP) ratio for different organs (leaves, stems, and roots) of annual (a-c) and perennial plants (d-f). L, S, and R represent leaves, stems, and roots, respectively.

## Supplementary Tables

**Table S1.** Information on 30 dominant plant species sampled in the riparian zone of the Three Gorges Reservoir in China.

| Species | Family | Genus | Life form |
| --- | --- | --- | --- |
| *Abutilon theophrasti* | Malvaceae | Abutilon | Annual herbs |
| *Aeschynomene indica* | Leguminosae | Aeschynomene | Annual herbs |
| *Alternanthera philoxeroides* | Amaranthaceae | Alternanthera | Perennial herbs |
| *Artemisia selengensis* | Compositae | Artemisia | Perennial herbs |
| *Arthraxon hispidus* | Gramineae | Arthraxon | Annual herbs |
| *Bidens tripartita* | Compositae | Bidens | Annual herbs |
| *Celosia argentea* | Amaranthaceae | Celosia | Annual herbs |
| *Conyza canadensis* | Compositae | Conyza | Annual herbs |
| *Cynodon dactylon* | Gramineae | Cynodon | Perennial herbs |
| *Cyperus michelianus* | Cyperaceae | Cyperus | Annual herbs |
| *Cyperus rotundus* | Cyperaceae | Cyperus | Perennial herbs |
| *Echinochloa crusgalli* | Gramineae | Echinochloa | Annual herbs |
| *Eclipta prostrata* | Compositae | Eclipta | Annual herbs |
| *Elymus dahuricus* | Gramineae | Elymus | Perennial herbs |
| *Hemarthria altissima* | Gramineae | Hemarthria | Perennial herbs |
| *Humulus scandens* | Moraceae | Humulus | Annual herbs |
| *Imperata cylindrica* | Gramineae | Imperata | Perennial herbs |
| *Leonurus sibiricus* | Labiatae | Leonurus | Annual herbs |
| *Melilotus officinalis* | Leguminosae | Melilotus | Annual herbs |
| *Mosla dianthera* | Labiatae | Mosla | Annual herbs |
| *Mosla scabra* | Labiatae | Mosla | Annual herbs |
| *Paspalum paspaloides* | Gramineae | Paspalum | Perennial herbs |
| *Polygonum chinense* | Polygonaceae | Polygonum | Perennial herbs |
| *Polygonum lapathifolium* | Polygonaceae | Polygonum | Annual herbs |
| *Rorippa indica* | Cruciferae | Rorippa | Annual herbs |
| *Saccharum spontaneum* | Gramineae | Saccharum | Perennial herbs |
| *Setaria viridis* | Gramineae | Setaria | Annual herbs |
| *Trifolium repens* | Leguminosae | Trifolium | Perennial herbs |
| *Xanthium sibiricum* | Compositae | Xanthium | Annual herbs |

**Table S2** Interactive effects of flooding, plant communities, soil nutrients, and other characteristics on plant carbon, nitrogen, and phosphorus stoichiometry and proposed interpretations.

| Effect | Proposed interpretation | References |
| --- | --- | --- |
| Flooding→Soil | Flooding may cause loss of nutrients from the soil, and water runoff and sediment deposition during flooding may lead to changes in soil density. | Ye et al., 2019 |
| Flooding→Plant community | Flooding may simplify plant community composition, resulting in reduced community height, cover, and species diversity. | Zheng et al., 2021 |
| Soil→Plant community | Soil is the basis for plant growth and has a profound effect on the composition, structure, and distribution of plant communities. Different types of soil conditions are suitable for different types of plants, and therefore the soil properties directly shape the characteristics of plant communities. | Ye et al., 2020; Zheng et al., 2021 |

**References**

Ye C., Butler O.M., Chen C.R., Liu W.Z., Du M., and Zhang Q.F. (2020). Shifts in characteristics of the plant-soil system associated with flooding and revegetation in the riparian zone of Three Gorges Reservoir, China. *Geoderma*. 361, 114015. doi:10.1016/j.geoderma.2019.114015.

Ye C., Chen C., Butler O.M., Rashti M.R., Esfandbod M., Du M., et al. (2019). Spatial and temporal dynamics of nutrients in riparian soils after nine years of operation of the Three Gorges Reservoir, China. *Sci. Total Environ*. 664, 841-850. doi:10.1016/j.scitotenv.2019.02.036.

Zheng J., Arif M., Zhang S., Yuan Z., Zhang L., Li J., et al. (2021b). Dam inundation simplifies the plant community composition. *Sci. Total Environ*. 801, 149827-149839. doi:10.1016/j.scitotenv.2021.149827.

**Table S3** Carbon, nitrogen, and phosphorus stoichiometric ratios of different organs in plants in China, globally, and in other different riparian zones.

| Regions | Organs | C | N | P | C:N | C:P | N:P |
| --- | --- | --- | --- | --- | --- | --- | --- |
| This study | Leaf | 386.65 | 19.31 | 5.27 | 16.15 | 191.70 | 5.56 |
|  | Stem | 404.02 | 11.23 | 4.81 | 26.98 | 273.72 | 4.60 |
|  | Root | 388.22 | 9.32 | 3.27 | 16.63 | 223.06 | 4.77 |
| China terrestrial plants  (Tang et al., 2018) | Leaf | 436.50 | 14.16 | 1.11 | 30.83 | 393.24 | 12.76 |
|  | Stem | 448.10 | 3.04 | 0.31 | 147.40 | 1445.48 | 9.81 |
|  | Root | 417.80 | 4.87 | 0.47 | 85.79 | 888.94 | 10.36 |
| China forests  (Tang et al., 2018) | Leaf | 463.70 | 14.02 | 1.25 | 33.07 | 370.96 | 11.22 |
|  | Stem | 468.60 | 2.40 | 0.30 | 195.25 | 1562.00 | 8.00 |
|  | Root | 453.10 | 3.95 | 0.45 | 114.71 | 1006.89 | 8.78 |
| China grasslands  (Tang et al., 2018) | Leaf | 396.9 | 16.09 | 1.27 | 24.67 | 312.52 | 12.67 |
| Riparian zone of Dan River (Yu et al., 2019) | Aboveground  (Leaf and Stem) | 422.14 | 22.33 | 2.25 | 18.90 | 187.62 | 9.92 |
|  | Root | 300.13 | 14.24 | 1.8 | 21.08 | 166.74 | 7.91 |
| Riparian zone of the Lijang River (Huang et al., 2019) | Leaf | 522.56 | 23.78 | 2.83 | 24.18 | 208.8 | 9.04 |
| Global flora  (Ma et al., 2018) | Leaf | 464 | 20.1 | 1.8 | 23.08 | 257.78 | 11.17 |

**References**

Huang D., Wang D., and Ren Y. (2019). Using leaf nutrient stoichiometry as an indicator of flood tolerance and eutrophication in the riparian zone of the Lijang River. *Ecol. Indic*. 98, 821-829. doi:10.1016/j.ecolind.2018.11.064.

Ma S., He F., Tian D., Zou D., Yan Z., Yang Y., et al. (2018). Variations and determinants of carbon content in plants: A global synthesis. *Biogeosciences*. 15, 693-702. doi:10.5194/bg-15-693-2018.

Tang Z., Xu W., Zhou G., Bai Y., Li J., Tang X., et al. (2018). Patterns of plant carbon, nitrogen, and phosphorus concentration in relation to productivity in China's terrestrial ecosystems. *Proc. Natl. Acad. Sci. U. S. A*. 115, 4033-4038. doi:10.1073/pnas.1700295114.

Yu M., Tao Y., Liu W., Xing W., Liu G., Wang L., et al. (2019). C, N, and P stoichiometry and their interaction with different plant communities and soils in subtropical riparian wetlands. *Environ. Sci. Pollut. R*. 27, 1024-1034. doi:10.1007/s11356-019-07004-x.
